# Ectopic expression of the apple nucleus-encoded thylakoid protein MdY3IP1 triggers early-flowering and enhanced salt-tolerance in *Arabidopsis thaliana*

**DOI:** 10.1186/s12870-018-1232-6

**Published:** 2018-01-20

**Authors:** Jian-Qiang Yu, Jia-Hui Wang, Cui-Hui Sun, Quan-Yan Zhang, Da-Gang Hu, Yu-Jin Hao

**Affiliations:** 1 National Key Laboratory of Crop Biology, College of Horticulture Science and Engineering, Shandong Agricultural University, Tai-An, Shandong 271018 China; 2 National Key Laboratory of Crop Biology, MOA Key Laboratory of Horticultural Crop Biology and Germplasm Innovation, College of Horticulture Science and Engineering, Shandong Agricultural University, Tai-An, Shandong 271018 China

**Keywords:** MdY3IP1, PSI complex, Floral transition, Salt tolerance, ROS, Sugar metabolism

## Abstract

**Background:**

The roles in photosystem I (PSI) assembly of the nucleus-encoded thylakoid protein Y3IP1 who interacts with the plastid-encoded Ycf3 protein that has been well-characterized in plants. However, its function and potential mechanisms in other aspects remain poorly understood.

**Results:**

We identified the apple *MdY3IP1* gene, which encodes a protein highly homologous to the *Arabidopsis* Y3IP1 (AtY3IP1). Ectopic expression of *MdY3IP1* triggered early-flowering and enhanced salt tolerance in *Arabidopsis* plants. MdY3IP1 controlled floral transition by accelerating sugar metabolism process in plant cells, thereby influencing the expression of flowering-associated genes. The increase in salt stress tolerance in MdY3IP1-expressing plants correlated with reduced reactive oxygen species (ROS) accumulation, and an increase in lateral root development by regulating both auxin biosynthesis and transport, as followed by enhancement of salt tolerance in *Arabidopsis*. Overall, these findings provide new evidences for additional functions of Y3IP1-like proteins and their underlying mechanisms of which Y3IP1 confers early-flowering and salt tolerance phenotypes in plants.

**Conclusions:**

These observations suggest that plant growth and stress resistance can be affected by the regulation of the *MdY3IP1* gene. Further molecular and genetic approaches will accelerate our knowledge of MdY3IP1 functions in PSI complex formation and plants stress resistance, and inform strategies for creating transgenic crop varieties with early maturity and high-resistant to adverse environmental conditions.

**Electronic supplementary material:**

The online version of this article (10.1186/s12870-018-1232-6) contains supplementary material, which is available to authorized users.

## Background

Growth and development of higher plants largely depends on the photosynthetic activity derived from cooperating photosystem I (PSI) and photosystem II (PSII) complexes [1–3]. PSI is a fundamental pigment-binding protein complex that functions in photosynthetic energy and electron-transfer processes; it uses energy absorbed from sunlight to drive electron transport from plastocyanin to ferredoxin, in cyanobacteria, algae, and plants [4–7]. In higher plants, PSI is protected from photodamage by a highly efficient antioxidant network that reduces the accumulation of dangerous reactive oxygen species (ROS), by PROTON GRADIENT REGULATION 5 (PGR5)-dependent processes, and by the photoinhibition of PSII [8–10]. Therefore, PSI is considered a robust photosystem.

PSI has a molecular mass of approximately 600 kDa, and is composed of two subcomplexes: the PSI core complex and the light-harversting complex I (LHCI). It has at least twelve core subunits in higher plants, including three peripheral (PsaC, PsaD, and PsaE) and nine membrane-intrinsic subunits (PsaA, PsaB, and PsaF-PsaL) [11–14]. Although PSI structure and composition are well characterized, little is known about complex assembly at the membrane [14–16]. Labeling experiments indicate that PSI assembly is fast [12, 13], and this makes it difficult to identify additional PSI assembly intermediates.

Although PSI assembly steps remain elusive, several auxiliary protein factors that mediate its biogenesis and assembly in the thylakoid membrane have been identified [16–18]. Two plastid-encoded proteins, Ycf3 (hypothetical chloroplast reading frame number 3) and Ycf4 (hypothetical chloroplast reading frame number 4), are essential for the assembly of the PSI complex [18–21]. The plastid-encoded Ycf3 protein also interacts with the nucleus-encoded thylakoid protein Y3IP1 (Ycf3-interacting protein) for PSI assembly in *Arabidopsis* and tobacco [17]. In addition, PPD1 and PSA2 associate with PYG7 are found to regulate the accumulation of PSI, and are involved in PSI biogenesis as well [22–25]. Interestingly, novel PSI assembly factors are constantly discovered, including thylakoid membrane-bound FtsH proteases and PSA3 [26, 27]. FtsH proteases is responsible for proper biosynthesis of PSI, while PSA3 encoding a protein on the stromal face of the thylakoid membrane promotes the interaction between PSI and assembly factor PYG7 [26, 27].

The *Arabidopsis y3ip1* mutant had growth retardation, delayed development, light-green leaf color, and reduction of PSI accumulation phenotypes [17]. Furthermore, *Y3IP1* overexpression increased the tolerance to salinity and oxidative stresses in *Arabidopsis*, leading the gene to be named *CHLOROPLAST PROTEIN-ENHANCING STRESS TOLERANCE* (CEST) [28]. Despite these phenotypes, AtY3IP1 function in PS1 assembly and roles in other biological processes remain unknown. In this study, we isolated the apple *MdY3IP1*, a gene with high homology to the *Arabidopsis Y3IP1*. Ectopic expression of *MdY3IP1* triggered early-flowering and enhanced salt tolerance phenotypes in *Arabidopsis thaliana*. Further analysis found that MdY3IP1 controlled floral transition by accelerating sugar metabolism process in plant cells, and, consequently, altering the expression of flowering-associated genes. MdY3IP1 expression reduced reactive oxygen species (ROS) production and promoted lateral root development by regulating both auxin biosynthesis and transport in salt stressed plants.

## Results

### Ectopic expression of the apple chloroplast-localized protein MdY3IP1 leads to early flowering in *Arabidopsis*

**Fig. 1:**
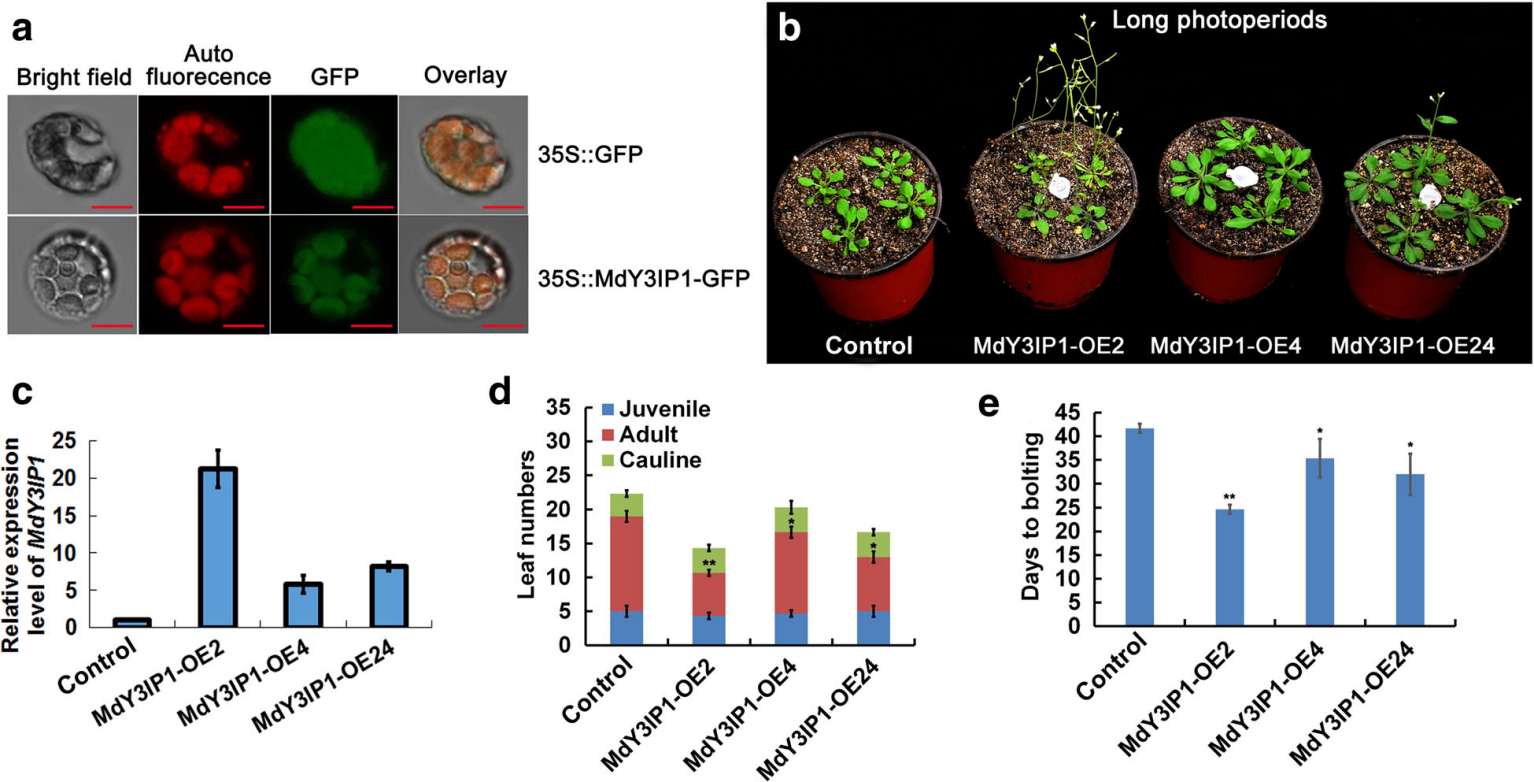
Early flowering phenotype in the *MdY3IP1*-expressing transgenic *Arabidopsis* plants. **a** Subcellular localization of MdY3IP1. pCaMV35S::MdY3IP1-GFP was transiently expressed in protoplasts of apple leaves. pCaMV35S::GFP was used as a negative control. Bars = 100 μm. **b** Flowering phenotype of the *MdY3IP1* transgenic *Arabidopsis*. 4-week-old plants grown in soil under long days (LDs) were photographed. Note: *Arabidopsis* plants transformed with an empty vector serve as the control. **c** Expression of *MdY3IP1* in the control and *MdY3IP1* transgenic plants by qPCR assay. Note: *Arabidopsis* plants transformed with an empty vector serve as the control. **d, e** Determination of leaf number (**d**) and of days to bolting (**e**). Approximate 20 plants grown under LDs were counted and averaged in each assay. Note: In **d, e**, data are shown as the mean ± SE, based on more than nine replicates. Statistical significance was determined using Student’s *t* test. *P < 0.01; **P < 0.001

We identified and cloned the apple *MdY3IP1* gene (Accession number: MDP0000930948) (Additional file 1), which encodes a protein highly homologous to the *Arabidopsis* nucleus-encoded chloroplast-localized AtY3IP1 [17]. To determine the subcellular localization of MdY3IP1, we constructed and transiently expressed pCaMV35S::MdY3IP1-green fluorescent protein (GFP) fusion vectors in protoplasts isolated from apple leaves. A pCaMV35S::GFP plasmid was used as a negative control. The subcellular localization of pCaMV35S::GFP and pCaMV35S::MdY3IP1-GFP fusion proteins was determined using Laser Confocal Microscopy (LCM). The GFP signal of pCaMV35S::MdY3IP1-GFP was enriched at regions that overlapped with chlorophyll auto-fluorescence in the chloroplasts, whereas pCaMV35S::GFP localized throughout the whole cells (Fig. 1a). These results suggest that MdY3IP1 is a chloroplast-localized protein.

To characterize *MdY3IP1*, we stably transformed *Arabidopsis thaliana* plants with a construct constitutively expressing *MdY3IP1* (*35S::MdY3IP1*-*myc*). An “empty vector (*35S–myc*)” version was used as the negative control. After screening for homozygous seeds, three transgenic lines (MdY3IP1-OE2, MdY3IP1-OE4, and MdY3IP1-OE24) were isolated (Fig. 1b). Analysis of transcript levels by qPCR demonstrated that *MdY3IP1* was successfully expressed in all three transgenic *Arabidopsis* lines (Fig. 1c), while absent in the negative control. Subsequently, we analyzed flowering-related phenotypes of the transgenic plants under long-day and short-day conditions. The number of rosette leaves at flowering was significantly reduced in *MdY3IP1* transgenic plants compared to the control (Fig. 1d). Additionally, plants from all three *MdY3IP1* transgenic lines bolted earlier than the control (Fig. 1e; Additional file 2).

Taken together, these results indicate that ectopically expressing the chloroplast-localized apple protein MdY3IP1 accelerates floral transition, leading to an early flowering phenotype in *Arabidopsis* plants.

### MdY3IP1 alters the expression of flowering-associated genes by improving photosynthetic derived sugar metabolism

**Fig. 2:**
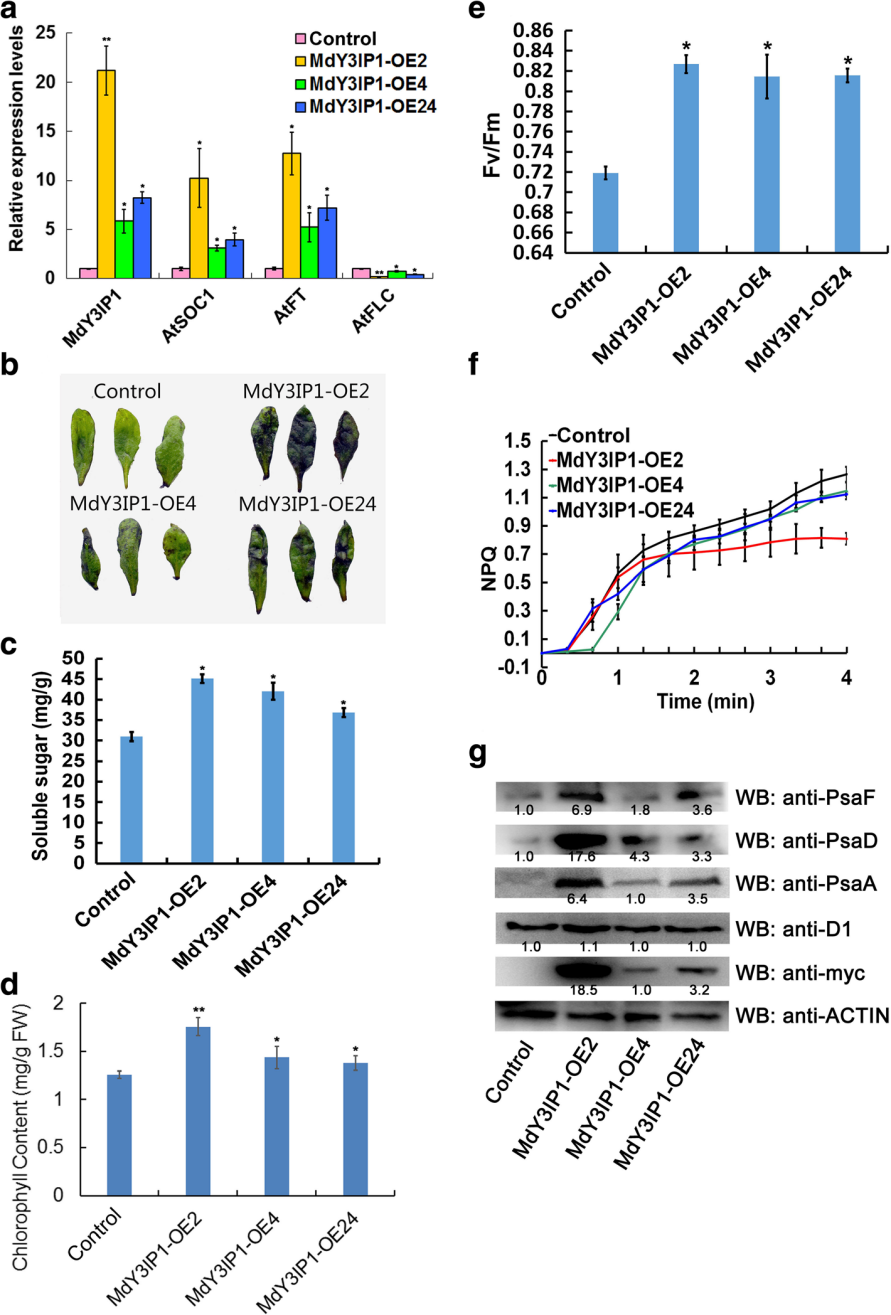
Photosynthetic sugar metabolism process is accelerated in *MdY3IP1* transgenic *Arabidopsis*. **a** qPCR analysis of *AtSOC1*, *AtFT* and *AtFLC* transcript levels in the control and *MdY3IP1* transgenic plants. The samplings were occurred around noon during the day time. **b** Starch staining of the control and *MdY3IP1* transgenic *Arabidopsis* plants. **c** Determination of soluble sugars in the control and *MdY3IP1* transgenic *Arabidopsis* plants. **d** Chlorophyll content in the leaves of control and *MdY3IP1* transgenic *Arabidopsis* plants. **e** Maximum quantum yield of PSII (Fv/fm) in the leaves of control and *MdY3IP1* transgenic *Arabidopsis* plants. **f** Induction and relaxation of NPQ monitored during dark-to-light transition (120 μmol photons m^− 2^ s^− 1^). Curves represents an average of six independent measurement. **g** Immunoblots of PSI core protein subunits including PsaA, PsaD, and PsaF, as well as PSII reaction center protein D1 in the control and *MdY3IP1* transgenic *Arabidopsis* plants. An anti-myc antibody was used to detected protein abundance of *MdY3IP1-myc* transgenic *Arabidopsis*. Anti-ACTIN antibody was used as a negative control. Note: In **a, c, d, e**, data are shown as the mean ± SE, based on more than nine replicates. Statistical significance was determined using Student’s *t* test. *P < 0.01; **P < 0.001

To further understand how MdY3IP1 regulates flowering in *Arabidopsis*, we examined the expression of various flowering time-related genes, in control and MdY3IP1-expressing plants. The transcripts of *AtSOC1* and *AtFT*, two positive regulators of photoperiodic flowering, were significantly increased in all three *MdY3IP1* transgenic *Arabidopsis* lines (Fig. 2a). Contrarily, the expression of *AtFLC*, a repressor of flowering, was reduced (Fig. 2a).

Various studies have shown that regulation of sugar metabolism contributes to photoperiodic flowering [29, 30]. Furthermore, *Y3IP1* plays a key role in photosynthetic carbohydrate synthesis by altering PSI accumulation [17]. Thus, we hypothesized that *MdY3IP1* controls flowering by altering starch and soluble sugar content in plants. To verify this hypothesis, we performed iodine-starch staining and UV-spectrophotometry analyses in transgenic *Arabidopsis* plants. Starch and soluble sugar levels were elevated in all three *MdY3IP1* transgenic lines compared to the control (Fig. 2b, c), supporting that *MdY3IP1*-expression increases sugar contents to influence flowering. We additionally determined the chlorophyll contents, the efficiency of photosynthetic electron transport, and the abundance of PSI core complex subunits in the transgenic plants. As expected, the chlorophyll content was significantly higher in *MdY3IP1* transgenic *Arabidopsis* plants than in the control (Fig. 2d). An increase in the F_V_/F_M_ (Maximum quantum yield of PSII photochemistry), Φ_I_ (Effective PSI quantum yield), and Φ_ND_ (PSI donor side limitation) was accompanied by a significant decrease of the NPQ (Non-photochemical quenching) in *MdY3IP1* transgenic *Arabidopsis* plants, especially MdY3IP1-OE2 (Fig. 2e, f; Table 1). There was no statistically significant difference in the amount of the PSII reaction center protein D1 between control and *MdY3IP1*-expressing plants (Fig. 2g). In contrast, the abundance of PSI core complex subunits, including PsaA, PsaD, and PsaF, was much higher in *MdY3IP1* transgenic *Arabidopsis* (Fig. 2g). Collectively, these results suggest that *MdY3IP1*-expression in *Arabidopsis* improves photosynthetic carbohydrate synthesis may be mainly by PSI accumulation, consequently altering the expression of flowering-associated genes and accelerating flowering.

**Table 1:** Functional characteristics of the thylakoid membrane of WT (col) and *MdY3IP1* (*MdY3IP1-OE2*, *MdY3IP1-OE4* and *MdY3IP1-OE24*) transgenic *Arabidopsis* plants. Values were measured from plants grown under moderate light intensities (120 μmol photons m^− 2^ s^− 1^)

Photosynthetic parameter	WT (col)	*MdY3IP1-OE2*	*MdY3IP1-OE4*	*MdY3IP1-OE24*
Fraction of oxidizable PSI, PM	0.12 ± 0.01	0.16 ± 0.01*	0.12 ± 0.01	0.13 ± 0.02
Effective PSI quantum yield, ФI	0.38 ± 0.02	0.45 ± 0.03*	0.41 ± 0.01*	0.46 ± 0.03*
PSI donor side limitation, ФND	0.52 ± 0.02	0.63 ± 0.03*	0.56 ± 0.01*	0.56 ± 0.02
PSI acceptor side limitation, ФNA	0.10 ± 0.01	0.11 ± 0.02	0.11 ± 0.02	0.10 ± 0.01
Effective PSI quantum yield, ФII	0.25 ± 0.03	0.24 ± 0.05	0.27 ± 0.03	0.30 ± 0.03
Yield of non-regulated non-photochemical energy loss, ФNO	0.33 ± 0.02	0.39 ± 0.07	0.34 ± 0.02	0.33 ± 0.01
Non-photochemical energy dissipation, ФNPQ	0.42 ± 0.01	0.32 ± 0.04*	0.39 ± 0.01*	0.37 ± 0.02*
Excitation pressure of PSII, 1-qP	0.40 ± 0.05	0.41 ± 0.02	0.42 ± 0.04	0.42 ± 0.01

The values are the means±SD, *n* = 6–9. Statistically significant differences comparing the *MdY3IP1* transgenic *Arabidopsis* plants to that of the corresponding WT (col) are marked with asterix (*). See text for details. WT, wild-type

### MdY3IP1 expression enhances salt tolerance by reducing ROS accumulation

**Fig. 3:**
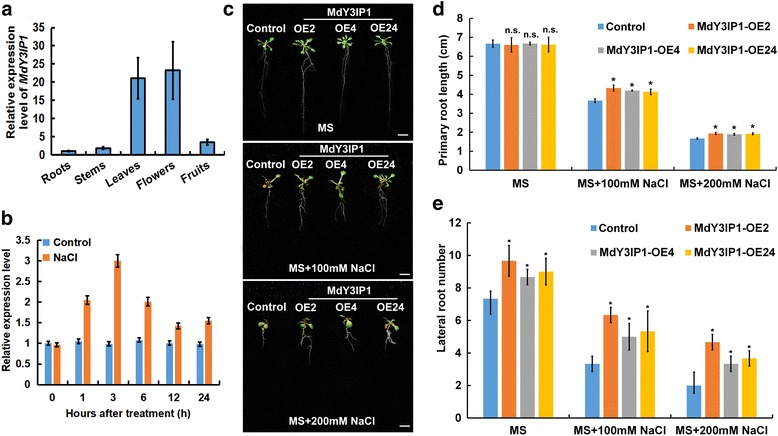
Ectopic expression of *MdY3IP1* enhances *Arabidopsis* tolerance to salt stress. **a** Expression of *MdY3IP1* in various apple tissues. Transcription levels are expressed relative to the level of transcripts in apple roots, which are arbitrarily set at 1. The data are represented by the mean value ± SD for triplicate values. **b** Expression level of *MdY3IP1* under 100 mM NaCl. ‘Gala’ cultivar plantlets were treated with 100 mM NaCl, and samples were collected at 0, 1, 3, 6, 9, 12, and 24 h after treatment for the expression analysis. **c** Phenotype of the control and *MdY3IP1* transgenic *Arabidopsis* under salt stress. *Arabidopsis* seeds were separately sowed to the MS medium. Subsequently, 5-day-old seedlings were transferred to new MS medium supplemented with 0, 100 and 200 mM NaCl, respectively. The photographs were taken at 20 days after transferring. Bars = 1 cm. **d, e** Primary root length (**d**) and lateral root numbers (**e**) of the control and *MdY3IP1* transgenic *Arabidopsis* plants as indicated in (**c**). Note: In **d, e**, data are shown as the mean ± SE, based on more than nine replicates. Statistical significance was determined using Student’s *t* test. n.s., *P* > 0.01; *P < 0.01

We analyzed the transcript levels of *MdY3IP1* in various apple organs. *MdY3IP1* was highly expressed in leaves and flowers, but detected at lower levels in the roots, stems, and fruits (Fig. 3a). Additionally, we investigated changes in *MdY3IP1* expression in apple plantlets exposed to various abiotic stresses, including high salinity (100 mM NaCl), low temperature (4 °C), oxidative stress (2% Polyethylene Glycol, PEG), and abscisic acid (100 μM ABA). *MdY3IP1* transcript levels were induced by all tested abiotic stresses, especially salt stress (Fig. 3b; Additional file 3). These results suggest MdY3IP1 is involved in abiotic stress responses.

To further understand the role of *MdY3IP1* in salt tolerance, we challenged *MdY3IP1*-expressing *Arabidopsis* plants with 100 mM or 200 mM NaCl. There was no obvious difference in primary root length between the control and *MdY3IP1* lines grown on MS medium without NaCl. However, in the plates supplemented with NaCl, *MdY3IP1* expression lead to a higher salt-tolerance and longer primary roots compared to the control (Fig. 3c, d). Interestingly, the number of lateral roots (LR) was always higher in *MdY3IP1* transgenic plants independent of growth conditions (Fig. 3c, e). These results indicated that *MdY3IP1* expression enhances salt tolerance in *Arabidopsis*.

**Fig. 4:**
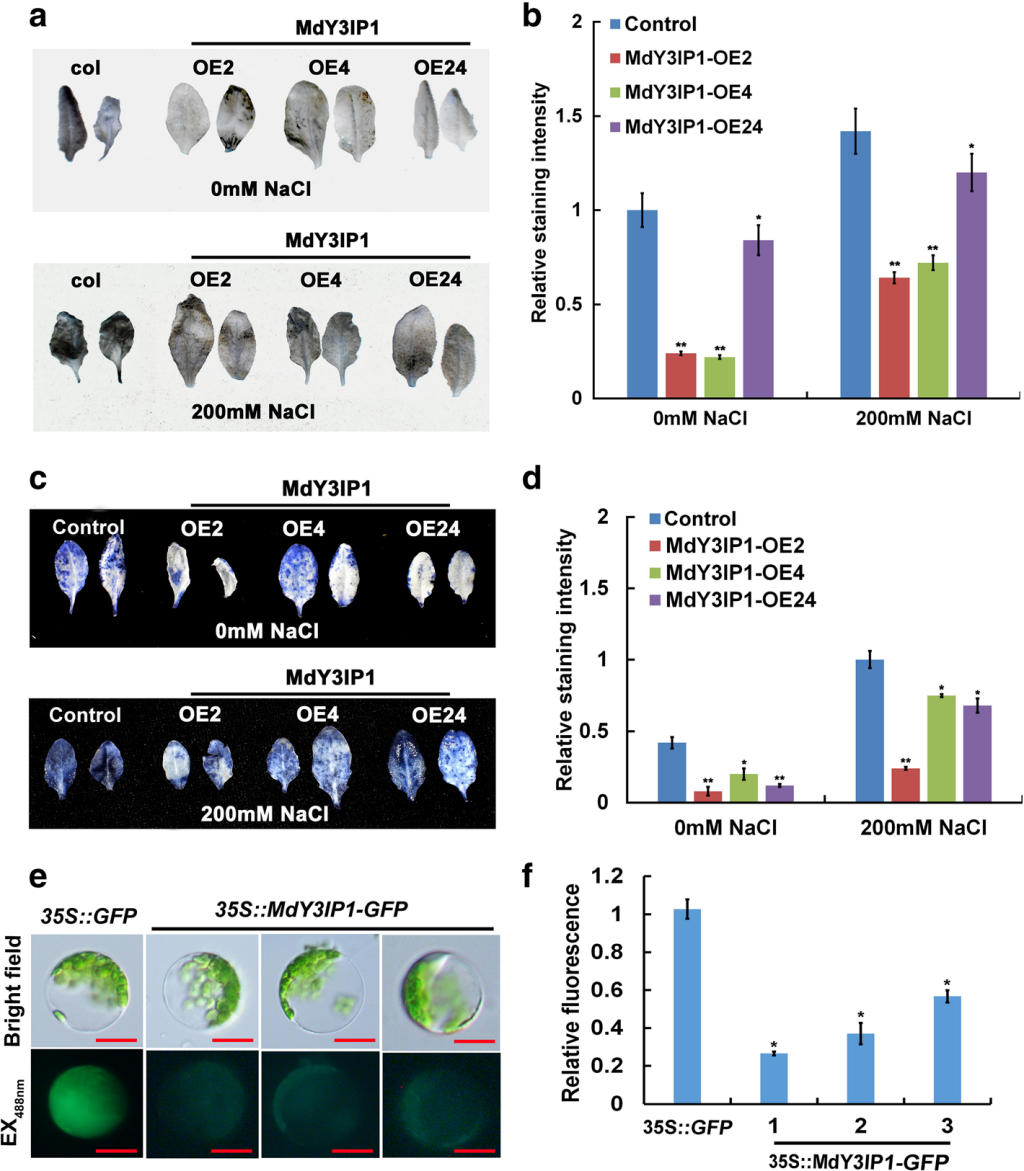
MdY3IP1 accumulates less ROS than the control in *Arabidopsis* leaves. **a** DAB staining for H_2_O_2_ in the leaves of the control and *MdY3IP1* transgenic *Arabidopsis* grown with or without a 200 mM NaCl. **b** DAB staining intensity as determined with imageJ software. **c** NBT staining for superoxide in the leaves of the control and *MdY3IP1* transgenic *Arabidopsis* grown with or without a 200 mM NaCl. **d** NBT staining intensity as determined with imageJ software. **e** DCFH-DA staining for H_2_O_2_ in the leaf protoplasts of the control and *MdY3IP1* transgenic *Arabidopsis* treated with or without 200 mM NaCl. Bars = 100 mm. **f** DCFH-DA staining intensity as determined with imageJ software. Three independent experiments were done with similar results, each with three replicates, and each replicate with 20 to 30 protoplasts. Note: In **b, d f**, data are shown as the mean ± SE, based on more than nine replicates. Statistical significance was determined using Student’s *t* test. *P < 0.01; **P < 0.001

It is widely known that high salinity induces oxidative stress [31, 32]. Therefore, we examined ROS accumulation in control and *MdY3IP1* transgenic *Arabidopsis* plants. We compared the production of H_2_O_2_ (DAB staining) and of superoxide (NBT staining) in control and *MdY3IP1*-expressing *Arabidopsis* leaves, and found that *MdY3IP1* plants accumulated less of ROS species in both salt-treated and untreated populations (Fig. 4a-d). DCFH-DA staining was also used to detect the accumulation of H_2_O_2_. The fluorescent signal corresponding to H_2_O_2_ was also much lower in *MdY3IP1*-expressing cells, confirming that *MdY3IP1* reduced ROS production in *Arabidopsis* (Fig. 4e, f). These results suggest that MdY3IP1-expression enhanced salt tolerance in *Arabidopsis* by reducing ROS accumulation in cells.

### MdY3IP1 promotes LR development by influencing local auxin biosynthesis and polar transport

**Fig. 5:**
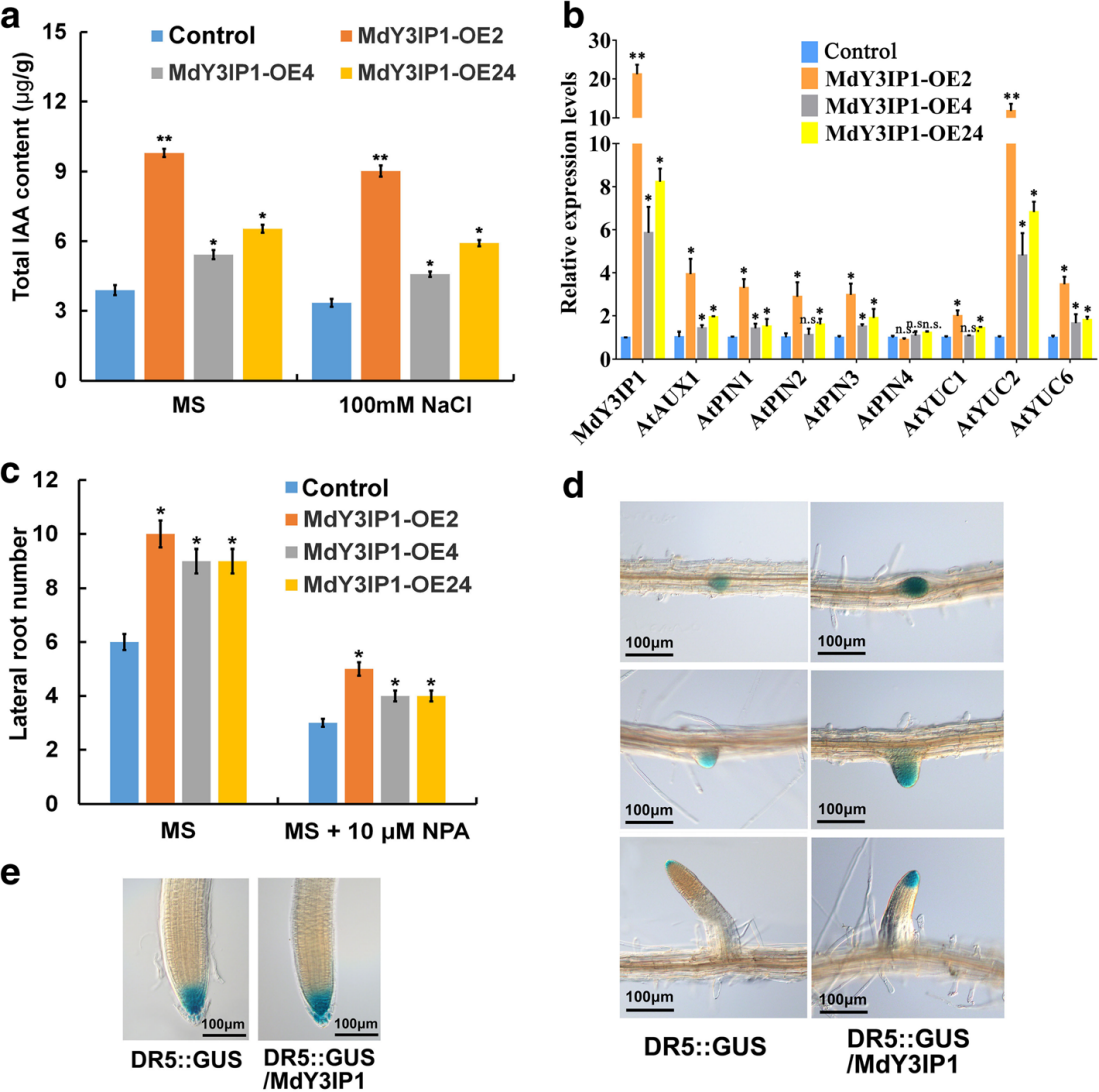
MdY3IP1 promotes auxin accumulation in lateral roots and lateral root primordia of transgenic *Arabidopsis* plants. **a** Total IAA content in the roots of 2-week-old control and *MdY3IP1* transgenic *Arabidopsis* plants. **b** Transcripts levels of auxin influx carriers (*AtAUX1*), efflux carriers (*AtPIN1*, *AtPIN2*, and *AtPIN3*), and biosynthetic *YUCCA* genes (*AtYUC1*, *AtYUC2* and *AtYUC6*), in control and *MdY3IP1* transgenic *Arabidopsis* by qPCR assay. **c** Effect of the auxin transport inhibitor NPA on LR initiation in control and *MdY3IP1* transgenic *Arabidopsis* plants. 5-day-old seedlings were transferred to MS medium supplemented with NPA (10 μM). After 10-days growth, the emerged LRs of 10 to 13 seedlings were counted. **d** Histochemical staining of GUS activity in LR primordia (top and middle panel) and LR (bottom panel) at three different stages. 10-day-old *Arabidopsis* plants expressing *DR5:GUS* in control (left) or *MdY3IP1* transgenic plants (right) were used for GUS staining for 8 h. Bars = 100 μm. **e** Histochemical staining of GUS activity in primary roots. 10-day-old *Arabidopsis* plants expressing *DR5:GUS* in control (left) or *MdY3IP1* transgenic plants (right) were used for GUS staining for 8 h. Bars = 100 μm. Note: In **a, b, c**, data are shown as the mean ± SE**,** based on more than nine replicates. Statistical significance was determined using Student’s *t* test. n.s., P > 0.01; *P < 0.01; **P < 0.001

Extensive research has shown that multiple hormonal pathways and environmental conditions influence LR initiation and development by regulating auxin homeostasis, biosynthesis, and transport [33]. Hence, we determined the total auxin content in control and *MdY3IP1* transgenic *Arabidopsis* plants. The *MdY3IP1*-expressing plants accumulated more auxin than the control whether it was on normal MS medium or on MS medium supplemented with 100 mM NaCl (Fig. 5a). Moreover, the transcript levels of auxin influx carriers (*AtAUX1*), efflux carriers (*AtPIN1*, *AtPIN2,* and *AtPIN3*), and biosynthetic *YUCCA* genes (*AtYUC1*, *AtYUC2*, and *AtYUC6*) were increased in *MdY3IP1*-expressing plants compared to the control (Fig. 5b). This implies that MdY3IP1 promotes LR development by regulating auxin biosynthesis and polar transport.

To further confirm this possibility, we grew seedlings on MS medium supplemented with the auxin transport inhibitor N-1-naphthylphthalamic acid (NPA). Application of 10 μM NPA significantly inhibited LR numbers in both control and *MdY3IP1* transgenic plants. However, LR numbers in *MdY3IP1* transgenic plants slightly increased compared to the control under this condition (Fig. 5c), whereas no significant difference in primary root length were observed between the control and *MdY3IP1* transgenic plants (Additional file 4). These results suggested that MdY3IP1 promotes only the LR development, and NPA could not completely eliminate the preferential LR development in transgenic plants.

As our results suggested that MdY3IP1 mediates LR development in an auxin-dependent manner (Fig. 5a, b), we examined endogenous auxin levels with the *DR5::GUS* reporter; this reporter localizes to regions with high auxin content. GUS staining revealed that the reporter was expressed in root tips of primary roots, as well as in LR and their primordia, independent of genotype (Fig. 5d, e). Interestingly, GUS staining was dramatically increased in emerged LR and LR primordia of *MdY3IP1* transgenic *Arabidopsis* (Fig. 5d). However, *MdY3IP1* did not alter *DR5::GUS* expression in the primary root tips (Fig. 5e). These results suggest that MdY3IP1 especially affects endogenous auxin levels in LRs.

In summary, these results support that *MdY3IP1*-expression promotes LR development by influencing auxin local biosynthesis and polar transport.

### Exogenous auxin application mimics the effect of *MdY3IP1*-expression in *Arabidopsis* roots

**Fig. 6:**
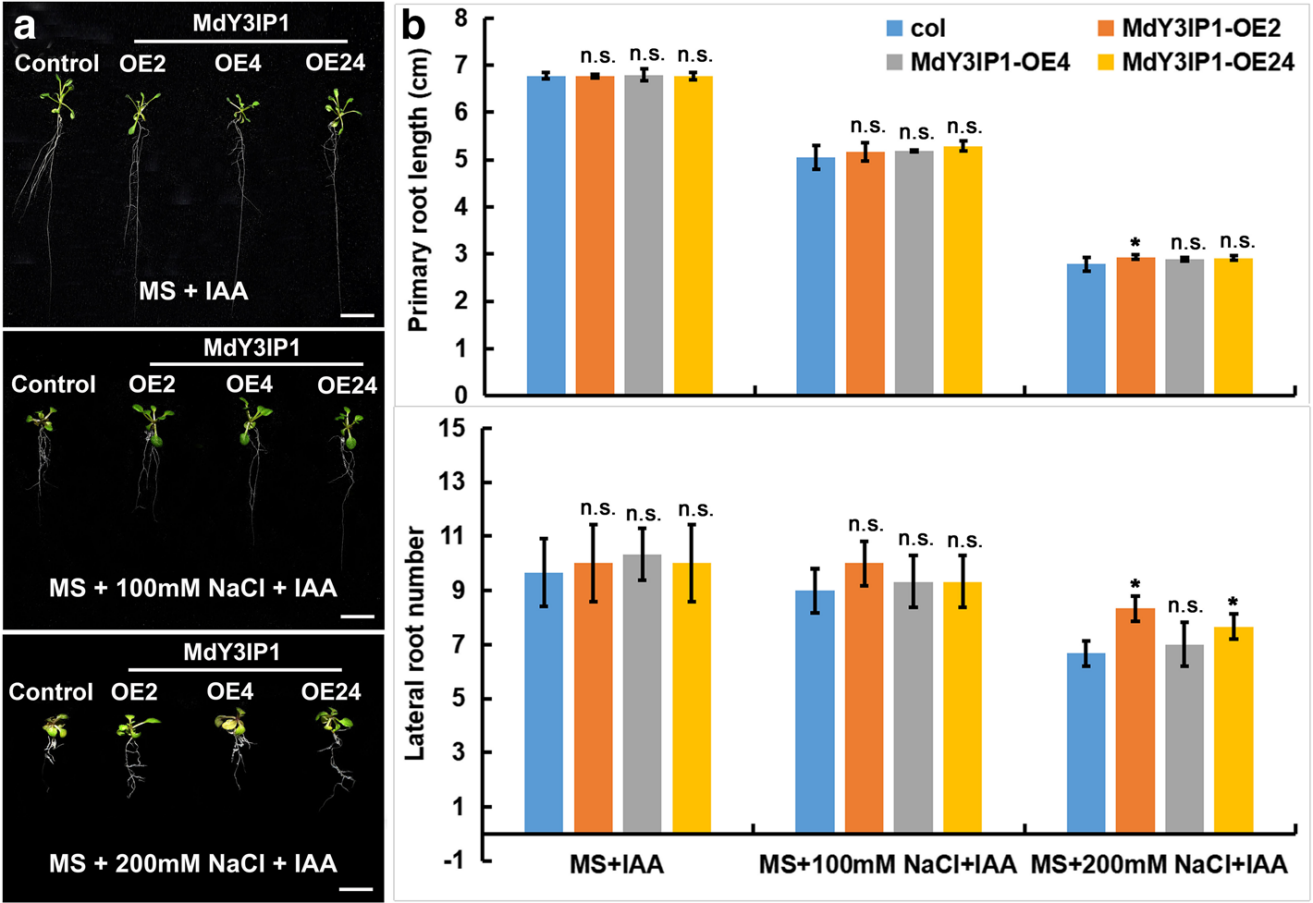
IAA application mimics the effect of *MdY3IP1*-expression in *Arabidopsis* roots. **a** Root architecture of control and *MdY3IP1* transgenic *Arabidopsis* plants grown on MS medium supplemented with 0, 100 and 200 mM NaCl, respectively. Bars = 1 cm. **b** primary root length and LR numbers of control and *MdY3IP1* transgenic *Arabidopsis* plants as indicated in (**a**). Note: In **b**, data are shown as the mean ± SE, based on more than nine replicates. Statistical significance was determined using Student’s *t* test. n.s., P > 0.01; *P < 0.01

IAA (Indole-3-acetic acid) is a form of auxin that occurs naturally and is commonly used in studies of auxin homeostasis, transport, and response during LR initiation and development [33]. To confirm the role of high auxin levels in the LR phenotype of *MdY3IP1*-expressing *Arabidopsis* plants, we included IAA (0.1 μM) in our growth medium. IAA application mimicked the effect of *MdY3IP1* expression in the LR developmental phenotype of the control and *MdY3IP1*-expressing *Arabidopsis* plants grown in MS media with or without NaCl (Fig. 6a, b). No significant difference was observed in primary root development when applying IAA (Fig. 6b). These results further support that MdY3IP1 affects primary and lateral root development under salt stress condition through its effects on auxin homeostasis.

## Discussion

PSI (plastocyanin-ferredoxin oxidoreductase), a participant of the photosynthetic electron transport chain, is one of the largest bioenergetics complexes known to date [6, 7, 13, 14]. Although PSI assembly steps are still not fully understood, several auxiliary protein factors have been proven to participate in that process [16–18]. The Y3IP1 protein specifically interacts with Ycf3 to promote PSI assembly in *Arabidopsis* and tobacco [17]. In this study, we identified the apple homolog of Y3IP1, MdY3IP1, and showed that ectopically expressing it in *Arabidopsis* lead to early flowering and enhanced salt tolerance phenotypes. These findings provide insights into new functions and potential additional mechanisms of Y3IP1 action in addition to its role in the PSI assembly.

**Fig. 7:**
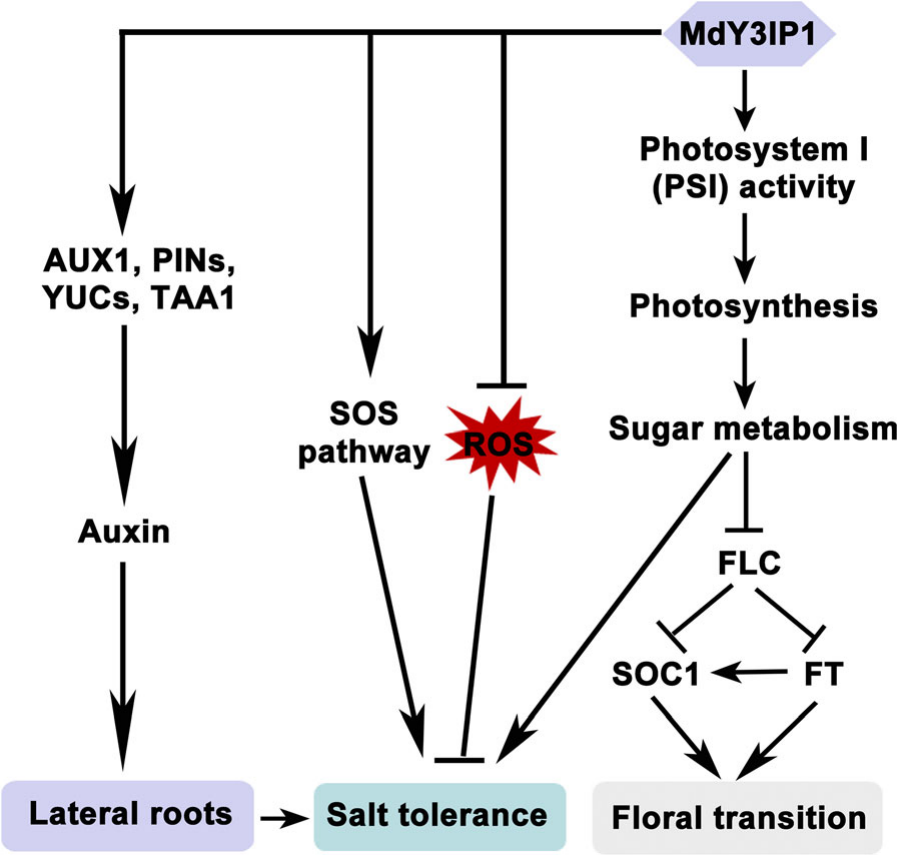
Working model for *MdY3IP1* function in the regulation of photoperiodic flowering and salt tolerance. In the current working model, *MdY3IP1* controls floral transition by altering the levels of sugar metabolism, and thereby influencing the expression of flowering-associated genes. *MdY3IP1*-expression reduces ROS production in plant cells, and promotes LR development by regulating auxin biosynthesis and transport. Additionally, it increases the expression of genes in the SOS pathway, and elevates the levels of sugar metabolism, leading to an increase in tolerance to salt stress in plants

Y3IP1 is an approximately 24 kDa nuclear-encoded protein that is post-translationally imported into chloroplasts. It closely associates with the thylakoid membrane [13, 17], most likely through a putative α-helical transmembrane domain on its C-terminal portion (Additional file 5) [13]. In *Arabidopsis*, *Y3IP1* overexpression strongly and specifically increased PSI accumulation [17], suggesting that Y3IP1 is required for PSI assembly. It is known that photosynthesis-derived sugar metabolism promotes flowering by regulating *FT* gene expression [29, 34]. Thus, it was not surprising that *MdY3IP1*-expression in *Arabidopsis* improved photosynthetic carbohydrate synthesis by PSI but not PSII accumulation, consequently altering the expression of flowering-associated genes including *FLC*, *SOC1* and *FT*, and causing an early flowering phenotype (Figs. 1, 2 and 7; Table 1). In addition, the *y3ip1* mutant phenotype in *Arabidopsis* suggested that Y3IP1 promotes plant growth and development [17]. This result was in line with our early flowering phenotype.

Plant growth and development are adversely affected by multiple stresses, which generate various types of dangerous cell-damaging ROS [35–37]. Plants have evolved different types of defense against the adverse effects of ROS, including production of antioxidant compounds and various kinds of defense proteins. For example, chloroplasts contain high concentrations of antioxidant molecules, like ascorbate and glutathione. Additionally, several types of antioxidant enzymes, including ascorbate peroxidase (APX) and superoxide dismutase (SOD), play critical roles in the scavenging of ROS [38, 39]. *MdY3IP1* expression was induced in apple plantlets by various abiotic stress treatments, including salinity, low temperature and drought stress (Fig. 3b; Additional file 3). Interestingly, *MdY3IP1*-expression in *Arabidopsis* enhanced salt tolerance by reducing ROS production (Fig. 4). Previous work showed that *Y3IP1* overexpression in *Arabidopsis* increased its tolerance to salt and oxidative stresses, leading to the gene named *CHLOROPLAST PROTEIN-ENHANCING STRESS TOLERANCE* (*CEST*) [28]. The improvement in salt tolerance was attributed to elevated PSI levels in *Y3IP1*-overexpressing plants [13].

It was previously reported that plant sugars play crucial roles during abiotic stress response [40]. Similarly, we speculate that *MdY3IP1*-expression increases salt tolerance by improving sugar metabolism (Fig. 7). We also found that *MdY3IP1*-expression reduced ROS accumulation and enhance salt tolerance (Figs. 4 and 7). Remarkably, salt overly sensitive (SOS) pathway genes, including *AtSOS1*, *AtSOS2*, and *AtSOS3*, were upregulated in *MdY3IP1*-overexpressing *Arabidopsis* (Additional file 6), implying that MdY3IP1 increases salt tolerance partly through the SOS pathway (Fig. 7).

The root system architecture (RSA) is plastic and dynamic, allowing plants to respond to various environmental stresses [41, 42]. RSA modifications in salt challenged plants include modulation of LR development by the auxin homeostasis-regulating transcription factor, WRKY46 [33]. Another example is the improved growth observed in *Trichoderma spp.-*treated *Arabidopsis* seedlings grown under salt stress, which correlates with enhanced root development, osmolite production, and Na^+^ elimination through root exudates [43]. Interestingly, expression of *MdY3IP1* in *Arabidopsis* promoted LR development by influencing local auxin biosynthesis and polar transport (Fig. 5). LRs initiate from cell divisions in the pericycle of the primary root. The root primordium forms a meristem and pushes its way through the cell layers to generate the LR [44]. The phytohormone auxin is known to be a key regulator of LR development. Altering its content, biosynthesis, distribution, or downstream signaling pathways deeply influences LR formation [45–47]. Disturbance of normal auxin distribution in LR founder cells using polar auxin transport inhibitor NPA is sufficient to completely block LR initiation [48]. We propose that phytohormones exert their effect on LR formation partially through the function of Y3IP1-like genes. Hence, *MdY3IP1*-expression in *Arabidopsis* may enhance root development through auxin-regulated pathways, leading to RSA changes and higher tolerance to salt stress. Our proposed work model describes how *MdY3IP1*-expression confers early-flowering and salt tolerance phenotypes to plants (Fig. 7).

Here, we showed that overexpression of *MdY3IP1* conferred early-flowering and salt tolerance phenotypes. These observations suggest that plant growth and stress resistance can be affected by the regulation of the *MdY3IP1* gene. Further molecular and genetic approaches will accelerate our knowledge of MdY3IP1 functions in PSI complex formation and plants stress resistance, and inform strategies for creating transgenic crop varieties with early maturity and high-resistant to adverse environmental conditions.

## Conclusion

In this study, we showed that MdY3IP1 encodes an apple protein highly homologous to the *Arabidopsis* Y3IP1 (AtY3IP1) PSI assembly factor. Ectopic expression of MdY3IP1 triggered early flowering and enhanced salt tolerance phenotypes in *Arabidopsis* plants. Further analyses found that MdY3IP1 controlled floral transition by improving photosynthetic derived sugar metabolism in plant cells, thereby influencing the expression of flowering-associated genes. Additionally, MdY3IP1 enhanced salt stress tolerance, by reducing ROS levels, and promoting LR development through auxin biosynthesis and transport pathways. Our observations confirm that plant growth and stress resistance are affected by the regulation of *Y3IP1*-like genes. It accelerates our knowledge of Y3IP1 functions in PSI complex formation, plant stress resistance, and growth/development by more molecular and genetic approaches, and provides strategies for creating genetically modified crops with early maturity and high-resistant to stress.

## Methods

### Plant materials, growth conditions, and treatments

*Arabidopsis thaliana* ecotype ‘Columbia’ (col) was used in this study. *Arabidopsis* seeds were surface-sterilized for 5 min in 75% alcohol and 20 min in 10% sodium hypochlorite, then washed six times with sterile water. Seeds were plated on Murashige and Skoog (MS) solid medium containing 1% (*w*/*v*) sucrose and 0.7% (w/v) agar. After vernalizing at 4 °C for 3 days, seeds germinated at 22 °C under a 16-h light/8-h dark photoperiod. Subsequently, 2-week-old seedlings were transferred to soil for further studies.

For the N-1-naphthylphtha-lamic acid (NPA) treatment, 5-day-old seedlings were transferred on MS supplemented with 5 μM of the chemical (Greyhound Chem Service, Birkenhead, UK). These plates were placed vertically, and grown at 22 °C under a 16-h light/8-h dark photoperiod for 10 days.

### Subcellular localization

The full-length ORF of *MdY3IP1* was cloned and inserted in a T vector (1258) digested with XcmI (NEB, Beijing) using the T4 ligase (TaKaRa, Japan). The *pCaMV35S::MdY3IP1-GFP* recombinant plasmid was transiently transformed into protoplasts extracted from young apple leaves. The protoplast extraction methods were described before [49]. Images were obtained using a confocal laser scanning microscope (LSM510; Carl Zeiss, Oberkochen, Germany). GFP signals were collected using an emission filter of BP505–530 nm, with excitation at 488 nm, and autoflorencence red signals (MitoTracker stain) were obtained using BP 585–615 nm, with excitation at 543 nm. 

### Quantitative RT-PCR (qPCR) assays

Total RNA extraction from the *Arabidopsis* plants indicated in each qPCR assay was performed using the RNeasy plant mini-prep kit (TIANGEN, Beijing) according to the manufacturer’s instructions. After digestion with DNase I (TaKaRa, Dalian), 1 μg purified RNA was converted into single stranded cDNA using the M-MLV reverse transcriptase (TaKaRa, Dalian). qPCR reactions were previously described [49]. The housekeeping gene *AteIF4a* was used as control. More than 9 biological replicates were performed for each experiment. The primers used for qRT-PCR are listed in the Additional file 7.

### Heterologous transformation of *MdY3IP1* into *Arabidopsis*

For *Arabidopsis* transformation, the *35S–myc* and *35S::MdY3IP1-myc* recombinant plasmids were introduced into ecotype Columbia (Col-0) using the floral dip method [50]. The *Agrobacterium* strain used was GV3101. After two generation of selection in hygromycin (30 mg/L), seeds of screened transgenic plants were harvested. Homozygotes were identified with a qPCR assay. Homozygous transgenic seeds from individual plants were used in further experiments.

### Isolation of thylakoids and immunoblotting assays

Thylakoid proteins of control and *MdY3IP1* transgenic *Arabidopsis* were isolated from leaves using published procedures [51]. For immunoblotting assays, samples were normalized to chlorophyll, electrophoretically separated on an SDS-polyacrylamide gels [52], and transferred to Hybond-P polyvinylidene difluoride membranes (GE Healthcare) using standard protocols. Immunoblot detection was performed with specific antibodies using the ECL PLUS system (Signa VH/i; GE Healthcare, Waukesha, WI, USA). Polyclonal antibodies against ACTIN, myc, PsaA, PsaD, PsaF, and D1 were prepared from rabbit by the Abmart Company (Shanghai, China). Immunoblotting assays were described before [53].

### Measurement of chlorophyll content and chlorophyll fluorescence difference absorption spectroscopy

Fresh *Arabidopsis* leaves (1.0 g) were homogenized with a plant tissue homogenizer in 20 ml of 95% ethanol (*v*/v in ddH_2_O). The homogenized samples were then centrifuged at 12,000 g for 10 min at 4 °C. The supernatants were diluted 10-fold using 95% ethanol. Chlorophyll content was measured by a UV/visible spectrophotometer (OPTIZEN POP, Mecasys). An SPDA-502 chlorophyll meter (KONICA MINOLTA) was used for direct measurements.

Chlorophyll fluorescence was recorded with a pulse amplitude modulated fluorimeter (Dual-PAM-100; Heinz Walz) on intact *Arabidospis* plants, at room temperature. Plants were dark adapted for 1 h prior to determination of the maximum PSII quantum efficiency (Fv/fm) and non-photochemical quenching (NPQ). The contents of PSI were determined in thylakoid preparations as previously described [50]. PSI was quantified by determining the P700 difference absorption signal sat 830 to 870nmin in solubilized thylakoids, using the Dual-PAM instrument [54]. Measurement procedures and deconvolution methods were previously described [55, 56].

### Starch staining and determination of total soluble sugars

4-week-old leaves of control and *MdY3IP1* transgenic *Arabidopsis* were immersed in a solution of Lugol containing 0.2% iodine and 2% potassium iodine, and incubated for about 1 min. They were then washed with ddH_2_O and placed in a mounting solution (7.5 g gum arabic, 100 g chloroacetaldehyde, 5 mL glycerol, and 60 mL water). The Lugol-stained leaves were photographed with a standard camera.

For determining total soluble sugars content, fresh *Arabidopsis* leaves (0.2 g) were boiled in water for 30 min for extraction, and the sugar levels were determined using the anthrone reagent with glucose as a standard. The absorbance was read at 630 nm, and the sugar concentration was determined using a glucose standard curve.

### Measurement of ROS

2-week-old seedlings of control and *MdY3IP1* transgenic *Arabidopsis* were treated in liquid MS with or without 200 mM NaCl for 5 h before the seedlings were used for follow-up staining analysis. 3′, 3′-diaminobenzidine (DAB) and nitroblue tetrazolium (NBT) staining were conducted as described before [37]. For 2′,7′-dichlorodihydrofluorescin diacetate (DCFH-DA) staining to detect H_2_O_2_, protoplasts isolated from *Arabidopsis* leaves were incubated in a buffer containing 50 mM DCFH-DA (Sigma-Aldrich) and 20 mM K-phosphate at pH 6.0 in darkness for 10 min. These protoplasts were then photographed using a confocal laser scanning microscope (LSM510; Carl Zeiss, Oberkochen, Germany) with an excitation at 488 nm. The intensity of the fluorescent signals was quantified using the ImageJ software.

### Histochemical staining of GUS activity

GUS activity staining was conducted as described previously [57]. After incubating the plant material at 37 °C for 8 h in the dark, individual representative seedlings were photographed with a confocal laser scanning microscope (Zeiss LSM 510 META, Jena, Germany). A total of 20 to 30 *Arabidopsis* plants per genotype were imaged in this experiment.

### Auxin content measurement

2-week-old seedlings of the control and *MdY3IP1* transgenic *Arabidopsis* were harvested and ground to powder in liquid nitrogen. For each sample, approximate 0.1 g powder were resuspended in pre-cooling 80% methanol and mixed immediately. The samples were kept at 4 °C protected from light before 0.8 ng [^13^C]-IAA was added. IAA content measurement was performed using GC-QQQ (Agilent, 7000A).

### Statistical analysis

Samples were analyzed in triplicates, and the data expressed as the mean ± standard error (SE) unless noted otherwise. Statistical significance was determined using Student’s *t*-test. A difference at *P* ≤ 0.01 was considered significant (*), and *P* ≤ 0.001 was considered extremely significant (**).

## Additional files

Additional file 1: Figure S1. Analysis of the deduced amino acid of MdY3IP1. (DOC 1372 kb)

Additional file 2: Figure S2. Early flowering phenotype in the *MdY3IP1*-expressing transgenic *Arabidopsis* plants under short-days condition. (DOC 702 kb)

Additional file 3: Figure S3. Expression of *MdY3IP1* under 4 °C, 2% PEG and 100 μM ABA. (ZIP 292 kb)

Additional file 4: Figure S4. Effect of the auxin transport inhibitor NPA on primary root length in control and *MdY3IP1* transgenic *Arabidopsis* plants. (ZIP 476 kb)

Additional file 5: Figure S5. The deduced functional domains of MdY3IP1 protein. (DOC 44 kb)

Additional file 6: Figure S6. Expression levels of *AtSOS1*, *AtSOS2 and AtSOS3* in the control and *MdY3IP1* transgenic *Arabidopsis* plants by qPCR assay. (ZIP 275 kb)

Additional file 7: Table S1. The primers used for RT-PCR and qRT-PCR in this study. (PDF 18 kb)

## Abbreviations

CEST: Chloroplast protein-enhancing stress tolerance
LHCI: Light-harversting complex I
NPA: N-1-naphthylphtha-lamic acid
NPQ: Non-photochemical quenching
PGR5: PROTON GRADIENT REGULATION 5
PSI: Photosystem I
qRT-PCR: Quantitative reverse-transcription PCR
ROS: Reactive oxygen species
Y3IP1: Ycf3-interacting protein
Ycf3: Hypothetical chloroplast reading frame number 3
