# Supplementary material for: Ectopic expression of the apple nucleus-encoded thylakoid protein MdY3IP1 triggers early-flowering and enhanced salt-tolerance in Arabidopsis thaliana
Source: BMC Plant Biol. 2018 Jan 20;18:18. doi: 10.1186/s12870-018-1232-6 (PMC5775602; doi:10.1186/s12870-018-1232-6)
Supplement: Supplementary file 1 — Analysis of the deduced amino acid of MdY3IP1. (DOC 1372 kb) [file 12870_2018_1232_MOESM1_ESM.doc]

**Additional file 1**

**Figure S1.** Analysis of the deduced amino acid of MdY3IP1. (a) Alignment of amino acid sequences of AtY3IP1 (At5g44650)，OsY3IP1 (Os01g0795500) and three homologous apple MdY3IP1 sequences (MDP0000930948, MDP0000276884, MDP0000273117). The alignment was performed using DNAMAN. Red, blue, and dark indicate positions at which residues are identical, strongly similar, and weakly similar amino acid groups, respectively. The transmembrane region is surrounded by red frames. (b) Phylogenetic tree of AtY3IP1 (At5g44650)，OsY3IP1 (Os01g0795500) and three homologous apple MdY3IP1 sequences (MDP0000930948, MDP0000276884, MDP0000273117). The tree was constructed with MEGA5.0 by the Neighbor-Joining (NJ) method. Our cloned apple *MdY3IP1* gene in this study was indicated by the asterisk.

**
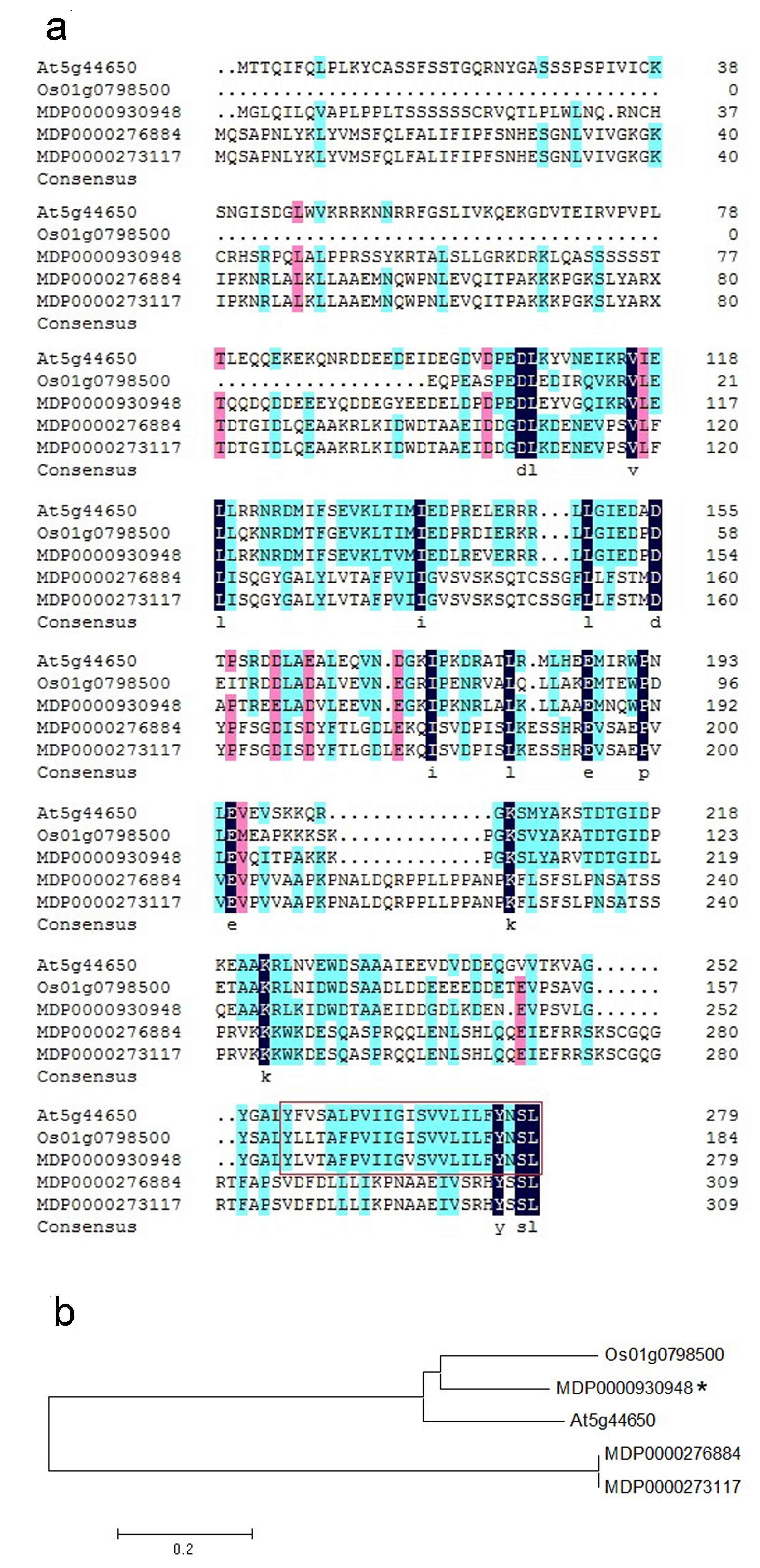
**
