# Supplementary material for: Ectopic expression of the apple nucleus-encoded thylakoid protein MdY3IP1 triggers early-flowering and enhanced salt-tolerance in Arabidopsis thaliana
Source: BMC Plant Biol. 2018 Jan 20;18:18. doi: 10.1186/s12870-018-1232-6 (PMC5775602; doi:10.1186/s12870-018-1232-6)
Supplement: Supplementary file 2 — Early flowering phenotype in the MdY3IP1-expressing transgenic Arabidopsis plants under short-days condition. (DOC 702 kb) [file 12870_2018_1232_MOESM2_ESM.doc]

**Additional file 2**

**Figure S2.** Early flowering phenotype in the *MdY3IP1*-expressing transgenic *Arabidopsis* plants under short-days condition. **a** Flowering phenotype of the *MdY3IP1* transgenic *Arabidopsis* under short-days condition. 4-week-old plants grown in soil under short days (SDs) were photographed. Note: *Arabidopsis* plants transformed with an empty vector serve as the control. **b** Determination of days to bolting. Approximate 20 plants grown under SDs were counted and averaged in each assay. Note: In **b**, data are shown as the mean ± SE, based on more than nine replicates. Statistical significance was determined using Student’s *t* test. *P < 0.01; **P < 0.001.

*
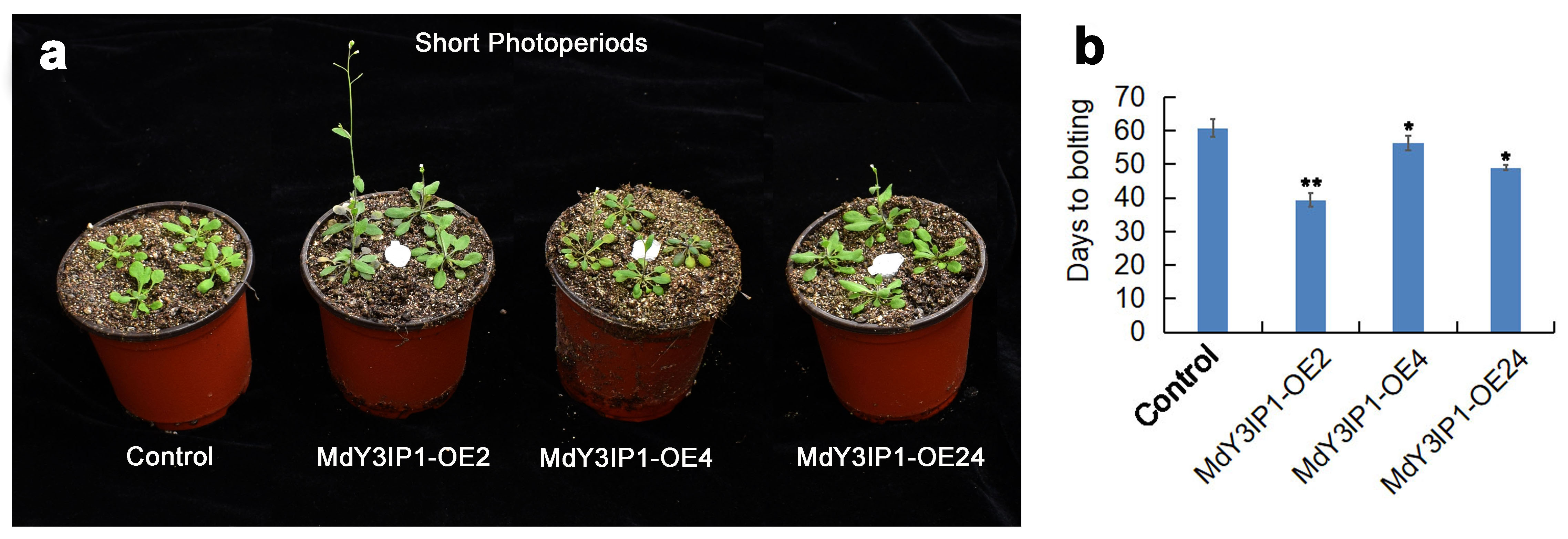
*
