# Supplementary material for: Ectopic expression of the apple nucleus-encoded thylakoid protein MdY3IP1 triggers early-flowering and enhanced salt-tolerance in Arabidopsis thaliana
Source: BMC Plant Biol. 2018 Jan 20;18:18. doi: 10.1186/s12870-018-1232-6 (PMC5775602; doi:10.1186/s12870-018-1232-6)

**Additional file 5**

**Figure S5.** The deduced functional domains of MdY3IP1 protein. The functional domains prediction was performed at the website http://smart.embl-heidelberg.de/.


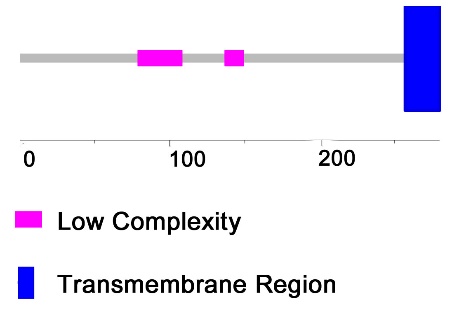

Supplement: Supplementary file 5 — The deduced functional domains of MdY3IP1 protein. (DOC 44 kb) [file 12870_2018_1232_MOESM5_ESM.doc]
