# Supplementary material for: Ectopic expression of the apple nucleus-encoded thylakoid protein MdY3IP1 triggers early-flowering and enhanced salt-tolerance in Arabidopsis thaliana
Source: BMC Plant Biol. 2018 Jan 20;18:18. doi: 10.1186/s12870-018-1232-6 (PMC5775602; doi:10.1186/s12870-018-1232-6)
Supplement: Supplementary file 7 — The primers used for RT-PCR and qRT-PCR in this study. (PDF 18 kb) [file 12870_2018_1232_MOESM7_ESM.pdf]

Table S1. The primers used for RT-PCR and qRT-PCR in this study.

Name	Primer sequences	Note
MdY3IP1-F(+A)	AATGGGTTTGCAAATTCTCC	Full length primers of *35S::MdY3IP1-myc*
MdY3IP1-R(+A)	CTGGAGCGAATTGTAAAACA	
MdY3IP1-F(qPCR)	AGGACCTGCGAGAAGTTGAG	qPCR primers
MdY3IP1-R(qPCR)	ACATCGTCCAATTCCTCCCT	
AtFLC-F(qPCR)	CCCTCTCCGTGACTAGAGCC	
AtFLC-R(qPCR)	TGGGAGAGTCACCGGAAGAT	
AtFT-F(qPCR)	GGTGGAGAAGACCTCAGGAA	
AtFT-R(qPCR)	GGTTGCTAGGACTTGGAACATC	
AtSOC1-F(qPCR)	TGAGGGGCAAAACTCAGATG	
AtSOC1-R(qPCR)	TCTTGCATATTGGAGCTGGC	
AteIF4a-F(qPCR)	TGACCACACAGTCTCTGCAA	
AteIF4a-R(qPCR)	ACCAGGGAGACTTGTTGGAC	
AtSOS1-F(qPCR)	TCGGCAGCATGGTTAATGTG	
AtSOS1-R(qPCR)	TTGGCTGAAACGAGACCTTGA	
AtSOS2-F(qPCR)	GAAACTGGAAAACATAATATGGAGGG	
AtSOS2-R(qPCR)	TCGCAGGACAAGTTTTGAGAAGTA	
AtSOS3-F(qPCR)	AATCCATCGCTCATCAAGAACA	
AtSOS3-R(qPCR)	CGGTTTATTTCCAAATCCTAGCTTAC	
