# Supplementary material for: Ectopic expression of the apple nucleus-encoded thylakoid protein MdY3IP1 triggers early-flowering and enhanced salt-tolerance in Arabidopsis thaliana
Source: BMC Plant Biol. 2018 Jan 20;18:18. doi: 10.1186/s12870-018-1232-6 (PMC5775602; doi:10.1186/s12870-018-1232-6)
Supplement: Supplementary file 3 — Expression of MdY3IP1 under 4 °C, 2% PEG and 100 μM ABA. (ZIP 292 kb) [file 12870_2018_1232_MOESM3_ESM.zip › Fig.S3.doc]

**Figure S3.** Expression of *MdY3IP1* under 4℃ (a), 2% PEG (b) and 100 μM ABA (c). Gala’ cultivar plantlets were treated with 4℃, 2% PEG and 100 μM ABA. Samples were collected at 0, 1, 3, 6, 9, 12, and 24 hours after treatment for the expression analysis. Samples with water treatment were used as the negative control. The data are represented by the mean±SD for the triplicate.
