# Supplementary material for: Ectopic expression of the apple nucleus-encoded thylakoid protein MdY3IP1 triggers early-flowering and enhanced salt-tolerance in Arabidopsis thaliana
Source: BMC Plant Biol. 2018 Jan 20;18:18. doi: 10.1186/s12870-018-1232-6 (PMC5775602; doi:10.1186/s12870-018-1232-6)
Supplement: Supplementary file 4 — Effect of the auxin transport inhibitor NPA on primary root length in control and MdY3IP1 transgenic Arabidopsis plants. (ZIP 476 kb) [file 12870_2018_1232_MOESM4_ESM.zip › Fig.S4.doc]

**Figure S4.** Effect of the auxin transport inhibitor NPA on primary root length in control and *MdY3IP1* transgenic *Arabidopsis* plants. 5-day-old seedlings were transferred to MS medium supplemented with NPA (10 μM). After 10-days growth, the primary root length of 10 to 13 seedlings were counted.
