# Supplementary material for: Ectopic expression of the apple nucleus-encoded thylakoid protein MdY3IP1 triggers early-flowering and enhanced salt-tolerance in Arabidopsis thaliana
Source: BMC Plant Biol. 2018 Jan 20;18:18. doi: 10.1186/s12870-018-1232-6 (PMC5775602; doi:10.1186/s12870-018-1232-6)
Supplement: Supplementary file 6 — Expression levels of AtSOS1, AtSOS2 and AtSOS3 in the control and MdY3IP1 transgenic Arabidopsis plants by qPCR assay. (ZIP 275 kb) [file 12870_2018_1232_MOESM6_ESM.zip › Fig.S6.doc]

**Figure S6.** Expression levels of *AtSOS1*, *AtSOS2 and AtSOS3* in the wild-type (WT) and *MdY3IP1* transgenic *Arabidopsis* plants by qPCR assay. Data are shown as the mean ± SE**,** based on more than nine replicates. Statistical significance was determined using Student’s *t* test. n.s., P > 0.01; *P < 0.01; **P < 0.001.
